# Metformin as a Metabolic Adjuvant to Amoxicillin: A Hypothesis on Synergistic Antibacterial Action

**DOI:** 10.34172/apb.025.43917

**Published:** 2025-09-03

**Authors:** Maher Monir. Akl, Amr Ahmed

**Affiliations:** ^1^Faculty of Medicine, Novosibirsk State University, Novosibirsk, Russia; ^2^The Public Health Department, Riyadh First Health Cluster, Ministry of Health, Riyadh, Saudi Arabia

## To Editor,

 Antibiotic resistance, driven by bacterial adaptability through genetic mutations and horizontal gene transfer, poses a critical global health challenge.^1^ Bacteria employ diverse resistance mechanisms, including beta-lactamase production, altered penicillin-binding proteins (PBPs), reduced membrane permeability, and efflux pumps, which lead to multidrug-resistant (MDR) and extensively drug-resistant (XDR) strains.^2,3^ These mechanisms increase mortality, prolong hospital stays, and raise healthcare costs, highlighting the urgent need for innovative strategies to enhance antibiotic efficacy and curb resistance.^4^

 Amoxicillin, a broad-spectrum beta-lactam antibiotic, inhibits bacterial cell wall synthesis by targeting PBPs, resulting in osmotic instability and cell lysis.^5^ It is effective against pathogens such as Streptococcus spp., Staphylococcus spp., Escherichia coli, and Haemophilus influenzae.^5^ However, resistance primarily through beta-lactamase enzymes that hydrolyze the beta-lactam ring, along with biofilms that limit antibiotic penetration compromises its effectiveness.^6,7^ Combining amoxicillin with beta-lactamase inhibitors like clavulanic acid can partially restore its activity, yet novel adjuvants remain essential to overcome complex resistance mechanisms.^8^

## Hypothesis

 We propose that metformin, traditionally used for glucose regulation, may serve as an adjuvant to amoxicillin, enhancing its antibacterial efficacy and potentially reducing resistance development through metabolic restriction and immunomodulation. While metformin lacks direct antibacterial action, its effects on host metabolism and immune responses may create an unfavorable environment for bacterial survival, thereby complementing amoxicillin’s bactericidal effect.

## Molecular mechanisms of metformin’s adjuvant potential

 Metformin activates AMP-activated protein kinase (AMPK), a central regulator of cellular energy homeostasis.^9^ AMPK activation shifts metabolism toward lipid oxidation and reduces glucose availability, particularly in inflamed tissues where bacteria exploit nutrient-rich conditions.^10^ This metabolic restriction may stress bacterial energy production, increasing susceptibility to amoxicillin’s cell wall-targeting effects.^11^ Additionally, metformin inhibits the mechanistic target of rapamycin (mTOR) pathway, reducing excessive immune cell proliferation and oxidative stress, which can otherwise promote bacterial adaptation.^12^

 Metformin also exerts immunomodulatory effects. By modulating the Treg/Th17 balance, it promotes anti-inflammatory cytokines (e.g., IL-10, TGF-β) and suppresses pro-inflammatory mediators (e.g., IL-6, IL-17).^13^ This shift reduces inflammation-driven nutrient and oxygen influx, limiting bacterial proliferation and enhancing biofilm disruption.^14^ By stabilizing the immune environment, metformin may decrease selective pressure for resistance mutations, supporting amoxicillin’s efficacy.

## Proposed synergy

 Metformin’s metabolic and immunological effects may amplify amoxicillin’s bactericidal action by creating an inhospitable environment for bacteria. Reduced glucose availability and inflammation hinder bacterial growth, while amoxicillin disrupts cell wall synthesis. This dual strategy could lower the necessary amoxicillin dose, thereby reducing side effects and resistance risk. Given metformin’s well-documented safety profile, it represents a promising candidate as an antibiotic adjuvant, though clinical dose optimization is required.^15^

## Therapeutic considerations

 For clinical application, metformin dosing may begin at 500 mg daily, titrated to 1,000–1,500 mg based on tolerance, aligning with its therapeutic range for AMPK activation.^15^ Amoxicillin dosing (500–1,000 mg every 8–12 hours) should follow standard infection-specific guidelines.^5^ Regular monitoring of kidney and liver function, glucose, and lactate levels is necessary to prevent complications such as lactic acidosis, particularly when metformin doses exceed 2,000 mg daily.^15^ Further preclinical and clinical studies are needed to validate this synergy and assess metformin’s role in non-diabetic patients.

## Conclusion

 We propose that co-administering metformin with amoxicillin could be a promising strategy to enhance antibiotic efficacy and mitigate resistance. Through metabolic restriction and immunomodulation via AMPK and mTOR pathways, combined with amoxicillin’s established bactericidal mechanism, this approach may improve treatment outcomes against MDR pathogens. Further research is essential to substantiate this hypothesis and optimize combination protocols.

## Competing Interests

 The authors declare no conflicts of interest.

## Ethical Approval

 Not applicable.
